# The Impact of Cross-linking Mode on the Physical and Antimicrobial Properties of a Chitosan/Bacterial Cellulose Composite

**DOI:** 10.3390/polym11030491

**Published:** 2019-03-13

**Authors:** Jun Liang, Rui Wang, Ruipeng Chen

**Affiliations:** 1 State Key Laboratory of Food Nutrition and Safety, Tianjin University of Science & Technology, Tianjin 300222, China; 13642198672@163.com; 2 College of Packaging and Printing Engineering, Tianjin University of Science & Technology, Tianjin 300222, China; chenruipeng2016@163.com

**Keywords:** chitosan, composite films, cross-linking, physical properties, bacteriostasis properties

## Abstract

The bacteriostatic performance of a chitosan film is closely related to its ionic and physical properties, which are significantly influenced by the mode of cross-linking. In the current work, chitosan with or without bacterial cellulose was cross-linked with borate, tripolyphosphate, or the mixture of borate and tripolyphosphate, and the composite films were obtained by a casting of dispersion. Mechanical measurements indicated that different modes of cross-linking led to varying degrees of film strength and elongation increases, while the films treated with the borate and tripolyphosphate mixture showed the best performance. Meanwhile, changes in the fractured sectional images showed a densified texture induced by cross-linkers, especially for the borate and tripolyphosphate mixture. Measurements of Fourier transform infrared showed the enhanced interaction between the matrix polymers treated by borate, confirmed by a slight increase in the glass transitional temperature and a higher surface hydrophobicity. However, the reduced antimicrobial efficiency of composite films against *E. coli*, *B. cinerea*, and *S. cerevisiae* was obtained in cross-linked films compared with chitosan/bacterial cellulose films, indicating that the impact on the antimicrobial function of chitosan is a noteworthy issue for cross-linking.

## 1. Introduction

An increased consumer demand for fresh, high quality foodstuff has given rise to an intense interest in the characteristics of active packaging materials that alter the conditions of the packaged food for extending shelf life, improving sensory qualities, or inhibiting the propagation of spoilage and pathogenic microorganisms [1]. In light of their availability, unique qualities, and eco-friendliness, the use of biopolymers within multiple food-packaging applications is quite beneficial [2,3]. Among the natural polymers used for bio-degradable packaging development, chitosan stands out on account of its intrinsic anti-microbe, solid mechanical strength, excellent barrier capacity, and biodegradability, as well as superior film-forming properties [4]. These unique properties have allowed for an exploration of its potential usage in the food industry as active edible food coatings or films in terms of improving food conservation by resorting to its antifungal and antibacterial ability [5].

In spite of the numerous benefits and original properties of chitosan, the existence of multiple amino groups and hydroxyl in the framework enhances its strong adhesion to water, and therefore chitosan films possess a high water swelling degree [6]. After over absorption of water, chitosan films become brittle, and are thus not applicable for packaging [7,8]. As a result, several techniques can be used to obtain the improved barrier and mechanical properties of chitosan films, including blending with polyvinyl alcohol, poly N-vinyl pyrrolidone, polyethylene glycol etc. [9,10,11].

Bacterial cellulose that is approximately less than 100 nm in diameter possesses unusual physical and mechanical properties [12,13]. Besides, bacterial cellulose and chitosan share similar structures and mutually complementary qualities, resulting in reinforced molecular interactions among polysaccharide chains. Compared with films of pure chitosan, chitosan/bacterial cellulose composite films exhibit more advantageous mechanical qualities, such as a rational thermal stability and a low O_2_ permeability [14]. Thereby, bacterial cellulose possesses great potential for being utilized as an agent of reinforcement in chitosan-based films, in terms of promising mechanical qualities and antimicrobial activity properties [15].

Another effective method for the improvement of films with desirable properties has emerged, namely, cross-linking treatment [16]. A polymer with an integrated network can be obtained by the cross-linking process, in which the polymer chains are interconnected by covalent or non-covalent linking [17]. In general, chitosan-based biomaterials are cross-linked by way of verified approaches, such as making use of cross-linking agents, the heat curing process, and ultraviolet irradiation or electron-beam [18,19]. There have been many investigations where chemically cross-linked chitosan-based biomaterials were treated with cross-linking agents, including glutaraldehyde, borate, formaldehyde, or 1,5- pentane-dial. In addition, researchers have investigated the physical property changes of films in relation to the degree of cross-linking [20]. A cross-linker is appropriate for biopolymer materials, in particular those obtained from proteins or carbohydrates, supplying reduced gas and water vapor permeability in food packaging materials [21,22]. Cross-linkers may make up for the intrinsic deficiencies in the barrier and mechanical properties of biopolymers, rendering them more applicable in comparison with petroleum-based counterparts [23]. Generally, remarkable mechanical properties, heat stability, and water resistance are obtained and the qualities of composite films might be controlled by means of adjusting the mode or the extent of cross-linking. However, a more detailed intermolecular force in the texture-property relationship cannot be achieved all the time.

**Scheme 1:**
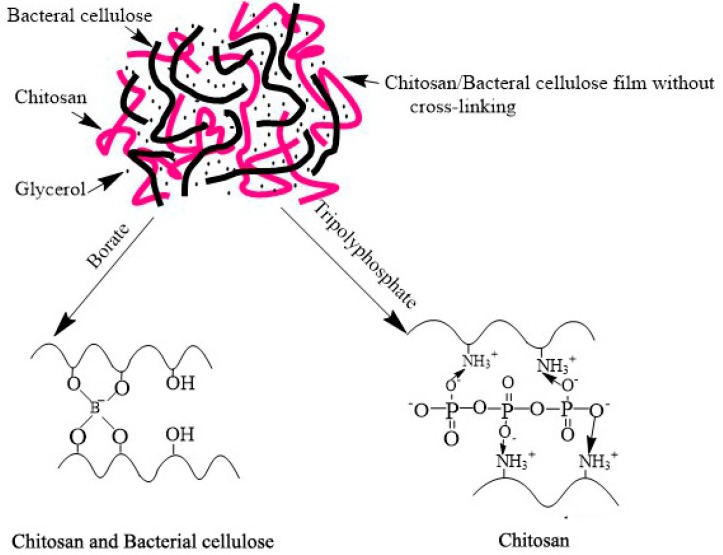
The rationale of cross-linking mode using borate and tripolyphosphate.

Therefore, in this study, a selective cross-linking method was used in the preparation of cross-linked chitosan/bacterial cellulose films to explore the impact of cross-linking mode on the macroscopic physical characterization along with the microphysical characterization of films simultaneously (Scheme 1). In view of the molecular characteristics of chitosan and bacterial cellulose, borate is able to cross-link chitosan and bacterial cellulose by forming hydroxyl groups with hydrogen bonds in matrix polymers [24]. Whereas by means of electrostatic interaction with the amino group, tripolyphosphate can produce a cross-linked structure with chitosan [25]. Natural cationic chitosan (CS) has been widely applied in constructing bactericidal coatings due to its contact-active disruption of the pathogen cytoplasmic membrane [26,27,28]. Thus, a noteworthy issue is how the reduction of hydroxyl or amino groups induced by cross-linking influences the antimicrobial property of chitosan, since iconicity is the origin of its contact-active function, despite the fact that the effects of some inserted nano or micron particles have been wildly explored [29,30,31]. It has been reported that when chitosan is immobilized onto a substrate, its antibacterial activities might be significantly reduced, proposed for the impeded ability of chitosan diffusion onto the cell membranes of microbes [32,33,34]. Nevertheless, chitosan is generally considered to be unable to enter the cell interior and the inhibitory effect is only exhibited on the cell surface due to its molecular weight. As a result, it is worth paying much more attention to the influence of the ionic property of chitosan, essential for its interaction with microbes, on the bacteriostasis abilities.

Hence, the present study aims to fabricate a chitosan/bacterial cellulose film treated by varied ways of cross-linking, verify the transformation of intermolecular forces in accordance with film mechanical properties, and clarify the antimicrobial ability changes induced by cross-linking. The composite films are also investigated using an electronic universal material test machine, electron scanning microscope (SEM), contact angle meter, water absorption and surface hydrophobicity tests, differential scanning calorimeter (DSC), and IR spectroscopy, as well as inhibition tests of bacteria, fungi, and yeast.

## 2. Materials and Methods

### 2.1. Materials

Chitosan (viscosity: above 400 mPa·s; average MW: 50–100 KDa; deacetylation: 85%) was purchased from Shanghai Macklin Biochemical Co., Ltd. (Shanghai, China). Bacterial cellulose (produced with *Acetobacter xylinum*; mean diameter: 50–100 nm; mean length: 10,000–20,000 nm; end group: free hydroxyl), was generously provided by Qihong Sci. and Tech. Co., Ltd. (Guilin, China). Glycerol, borate, and tripolyphosphate (Analytical grade purity >99%) were obtained from Damao Chemical Reagent Beijing CO., Ltd. (Beijing, China). *E. coli.* ATCC 25922, *B. cinerea* ATCC 30387, and *S. cerevisiae* BY 4743 yeast were obtained from the School of Biotechnology at Tianjin University of Science and Technology (Tianjin, China).

### 2.2. Sample Preparation

Chitosan/bacterial cellulose films of different weight ratios were prepared by means of solution casting in 10 cm×10 cm petri dishes according to the published method, but with slight modifications [35]. A total of 2 g of chitosan was dissolved in 100 mL 1% acetic acid solution at room temperature to form 2.0 w/v% polymer solutions. Then, 0.5 wt% bacterial cellulose solution was obtained by dissolving 0.5 g of bacterial cellulose powder in 100 mL DI water at room temperature and afterwards, the solution was sheared for 30 min with a Kesun JLL350-B2 blender. Following this, 100 mL mixture solutions made from chitosan and bacterial cellulose stock solutions of different volume ratios were added by a corresponding weight of plasticizer glycerol to obtain final bacterial cellulose/chitosan 0, 1/64, 1/32, 1/16, 1/8, and 1/4 with 40 wt% glycerol in dry films. The film-forming conditions were set at 50 °C and relative ambient humidity in a drier for 48 h. As for the cross-linked films, cross-linkers were added in the bacterial cellulose/chitosan 1/32 matrix with final concentrations of 4% borate (CB-b), 4% tripolyphosphate (CB-t) or 2% borate, and 2% tripolyphosphate mixture (CB-bt) in the dried films, respectively. The dry films (d CB-b, d CB-t, d CB-bt) were peeled off the dishes, and kept in storage at room temperature against the desiccants DrieRite, so as to maintain zero relative humility (RH) for at least one week before measurements. The average thickness of the film was 0.10 ± 0.01 mm. To explore the impact of water absorption on the mechanical characteristics of films, the wet films (w CB-b, w CB-t, w CB-bt) were obtained by placing dry films in a man-made temperature humidity chamber at 25 °C and 80% relative humidity in a MEA15004-014 (Jufu, Beijing, China) for 12 h.

### 2.3. Cross Sectional Structure

The fractured sectional image of sample films was examined by scanning electron microscopy (SEM). For fractured section structure images, films were frozen in liquid nitrogen and were broken later. The film fracture section coating with a gauzy film of gold (Au) was then put on double-sided Scotch tape installed on a Luminal specimen holder, and imaged under a Geol scanning electron microscope (SU-1510, Hitachi, Tokyo, Japan).

### 2.4. Mechanical Qualities

The elongation and tensile strength at the break of films was tracked on a 3396 electronic universal material test machine (Intron, Boston, MA, USA) according to China National Standard GB/T 1040-2006. Rectangular dry or wet specimens (1 cm × 10 cm) were incised using a precision double-blade cutter. Initial grip separation was fixed at 70 mm and the cross-head speed was set at 100 mm/min. A total of five measurements were calculated and averaged for each sample.

### 2.5. Thermal Studies

As a function of cross-linkers, the glass transition temperature was measured on an 8000 differential scanning calorimeter (DSC) (PerkinElmer, Boston, MA, USA). Films were put in pressure-tight DSC cells (about 8.5 mg of matter per cell). Highly purified nitrogen was channeled into the sample compartment and flushed for at least 30 min before measurement. The first scanning was performed ranging from −10 to 50 °C at the speed of 10 °C per minutes and the samples were kept at 50 °C for 30 min to expel the water residues in the films. After rapid cooling (10 °C/min to −10 °C), measurements were carried out during a second scanning at 10 °C/min from −10 to 220 °C. Glass transition temperature was determined in accordance with a half-variation in calorific capacity during phase transition.

### 2.6. Infrared Spectroscopy

FTIR measurements of the dry and wet films were carried out by way of a Nicolet 6700 spectrometer (Thermo, Waltham, MA, USA) at ambient temperature. Data were collected over 16 scans at a 16 cm^−1^ resolution and analyzed using Origin 8.6.

### 2.7. Contact Angle

The measurements of static water contact angle were performed by means of a VCA option Contact Angle Analyzer (Jike, Shanghai, China) under optimal conditions. The image was captured by a CCD camera right after a 10 μL water drop was deposited onto the film surface and from which a contact angle analysis could be performed. A total of five measurements were averaged for each sample.

### 2.8. Film Swelling Degree, Water Vapor Absorption, and Water Activity

The swelling degree (SD) of a given sample film was calculated as follows: firstly, a thoroughly dried sample was immersed in distilled water at 25 °C for 12 h; then, the samples were picked out and weighed after gentle wiping of the surface using absorbent paper. SD was measured as the percentage of initial film weight increase that occurred after swelling in water.

The water vapor absorption (WVA) of a given sample was measured as follows: sample films (10 cm × 10 cm) dried in desiccators were weighed (±1 mg) in glass dishes; then, they were put in a temperature humidity chamber at 25 °C and 80% relative humidity for 12 h. Water vapor absorption (WVA) was measured as the percentage of initial film weight growth that occurred during moistening and was determined on a wet basis.

The water activity (WA) of each dry and wet film was determined by a water activity detector (Dongxi Yike, Beijing, China).

Triplicate measurements of SD, WVA, and WA were determined individually, prepared as replicated experimental units.

### 2.9. Antimicrobial Testing

As far as the antimicrobial testing is concerned, the PE film as a blank (1 cm × 1 cm) was immersed in 10 mL liquid culture in a tube with a calibrated suspension of *E. coli* (1 × 10^6^ CFU), *B. cinerea* (5 × 10^5^ CFU), and *S. cerevisiae* (5 × 10^5^ CFU), respectively. After incubation at 37 °C with a constant shaking of 250 rpm for 12 h, 1 mL of the 100 times diluted microbe suspension was inoculated on an agar medium plate. Right after incubation at 37 °C for 24 h, the images of the agar plates were captured and the antimicrobial effects of the samples were evaluated on the basis of the colony count using the following equation:

*E*(%) = [(*A* − *B*)/*A*] × 100% (1)

where *A* = the number of viable microbe colony for the PE film (blank) in the plate and *B* = the number of viable microbe colonies for chitosan-based samples in the plate. A total of three measurements were averaged for each sample.

## 3. Results and Discussion

### 3.1. Mechanical Properties

**Figure 1:**
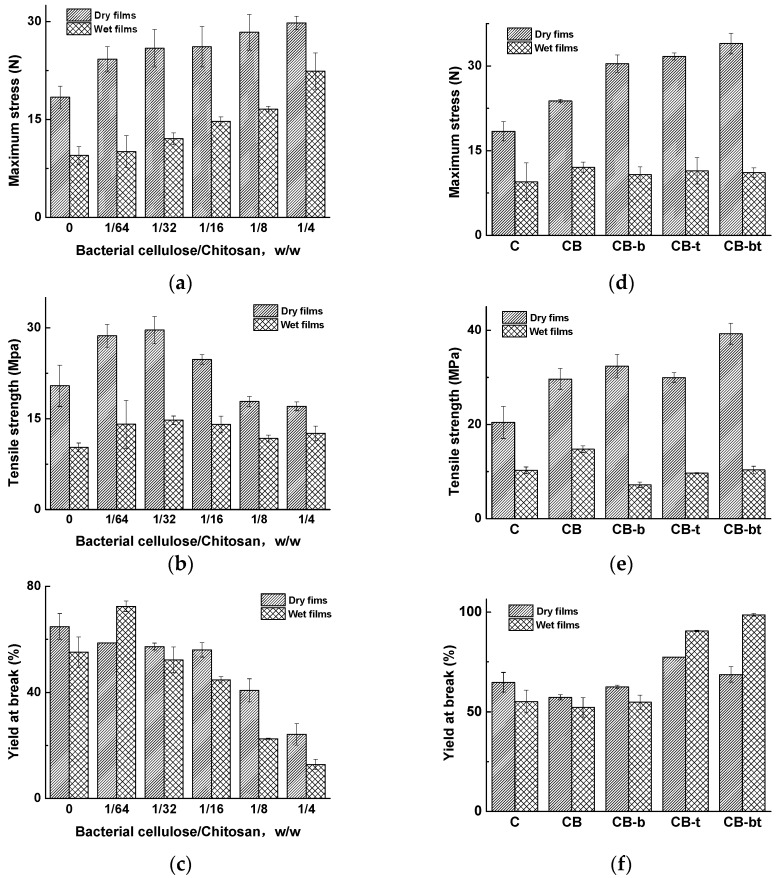
The impact of cross-linking treatment on the maximum strength, tensile strength, and yield at the break of dry and wet films of various ratios of bacterial cellulose/chitosan, chitosan (C), and 1/32 bacterial cellulose/chitosan (CB) treated with 4% borate (CB-b), 4% tripolyphosphate (CB-t), or a mixture of 2% borate and 2% tripolyphosphate (CB-bt). (**a**) maximum strength of Bacterial cellulose/Chitosan composite with varying weight ratio; (**b**) tensile strength of Bacterial cellulose/Chitosan composite with varying weight ratio; (**c**) yield at the break of Bacterial cellulose/Chitosan composite with varying weight ratio; (**d**) maximum strength 1/32 Bacterial cellulose/Chitosan composite with varying mode of cross linking; (**e**) tensile strength of 1/32 Bacterial cellulose/Chitosan composite with varying mode of cross linking; (**f**) yield at the break of 1/32 Bacterial cellulose/Chitosan composite with varying mode of cross linking.

The maximum stress, tensile strengths, and yield at break for all films in dry or wet conditions are summarized in Figure 1 using the average statistical data produced from the five analyzed specimens. Obviously, dry films possess a higher film strength, i.e., maximum stress and tensile strength, in contrast with wet films. Since the weight and shape of the films used are the same, the measured tensile force can truly reflect the mechanical qualities of the films. Regarding the films from chitosan reinforced with bacterial cellulose, it has been shown that the tensile strength firstly enhanced, and then declined with the concentration increase of bacterial cellulose (Figure 1b). This phenomenon might be related to the formation of intermolecular hydrogen bonds between the chemical groups in bacterial cellulose (–OH) and chitosan (–OH and –NH_2_), therefore restricting the motion of the matrix while promoting rigidity [36]. Consistently, it is also reported by Ciechańska that bacterial cellulose fibers form a three-dimensional network, which is hydrogen bonded to the glucan chain and staggered in chitosan to enhance the strength of the chitosan matrix [37]. The maximum reached the ratio of bacterial cellulose/chitosan 1/32. Accordingly, the bacterial cellulose/chitosan 1/32 film was selected for the subsequent experiments. As for CB-t and CB-bt films, however, the wet films manifested a better performance in yield at break (Figure 1f), indicating that the absorption of water induced an increase of elongation in matrices processed by tripolyphosphate and the borate/tripolyphosphate mixture. Studies by Salari et al. (2018) have shown that after the blending with 4% bacterial cellulose, tensile strength enhancement of chitosan film was close to 100%, but the performance of bacterial cellulose in our work is moderate, which might be due to the fact that we added 40 percent of glycerol and the films were plasticized and softened [38].

The impact of glycerol on the physical properties of polymer matrices has been extensively studied. Low-molecular weight compounds or diluents, acting as external plasticizers, are an integral part of polymeric systems. They serve to increase the flexibility and workability of the otherwise rigid neat polymers [39,40]. In the current work, the measured maximum strength and yield at the break for dry chitosan/bacterial cellulose with 40% glycerol are 20.4 ± 3.4 (MPa) and 64.8 ± 4.9 (%), respectively. Dhanavel and his staff have studied the chitosan film containing 40% glycerol. They obtained 9.4 MPa for the maximum strength and 66% for yield at the break [41]. Herein, the bacterial cellulose was proved to be able to effectively enhance the strength of chitosan films, improving its applicability in food packaging.

Concerning dry 1/32 bacterial cellulose/chitosan films with or without cross-linkers, the values of maximum strength (Figure 1d) and tensile strength (Figure 1e) range from 18.4 to 34.0 N and 20.4 to 39.0 MPa, respectively. Meanwhile, regarding film elongation (Figure 1f), namely yield at break, the value ranges from 57.3 to 77.4%. The introduction of cross-linkers caused an increase in both film strength and elongation, which could be attributed to the improvement of the interaction among matrix polymers [42]. Additionally, the cross-linker mixture performed best in terms of improving the film strength. A reasonable explanation for this is that cross-linker mixture induced a stronger intermolecular force network in the matrices, which was further studied by DSC and IR in the following section.

Chitosan and bacterial cellulose were highly absorbent. After being kept in an 80% humidity environment, a certain amount of water was absorbed in the films, which were described as wet films. In general, an increase in moisture uptake weakened the tensile strength of chitosan films [43]. Concerning the wet films, the values for maximum strength and tensile strength ranged from 9.5 to 11.4 N and 7.2 to 14.7 MPa, respectively. For yield at break, the value ranged from 52.2 to 98.6%. Unexpectedly, the introduction of moisture in wet films leads to a conspicuous decrease in film strength and an increase in film elongation. For films treated with tripolyphosphate or a mixture of borate and tripolyphosphate, the film elongation of wet films showed an obvious increase compared with that of dry films, and the water clearly displayed a plasticizer effect in the composite films, i.e., softening the chitosan/bacterial cellulose film. For films treated with borate, the film elongation of wet films showed an obvious decrease compared with that of dry films, and the water showed an antiplasticizer effect, i.e., stiffening the chitosan/bacterial cellulose films. The phenomenon of a plasticizer being able to exert a role of an antiplasticizer is well-known and has received increasing attention from food scientists and technologists in recent years [44,45,46]. It has been reported that small molecules like glycerol, water, etc. can exert different effects, depending on their concentration. In most cases, there is a critical element that marks the onset of a change in functionality from antiplasticizer to plasticizer, depending on the polymer matrices [47]. In this work, the various cross-linking modes lead to different roles of water in the chitosan/bacterial cellulose film.

### 3.2. Cross Sectional Fracture Structure

**Figure 2:**
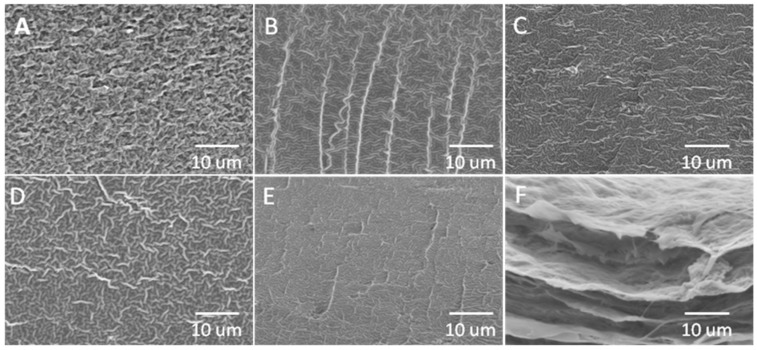
Fractured cross-sectional image of C (**A**), CB (**B**), CB-b (**C**), CB-t (**D**), CB-bt (**E**), and bacterial cellulose film imaged by SEM.

Cross sectional fracture structure of C, CB, CB-b, CB-t, CB-bt, and bacterial cellulose is shown as Figure 2A–F, respectively. Glycerol is known to enter polyhydroxylated polymers’ chain interior, disrupting inter-and intra-molecular interactions, rendering the polymer plasticized, as well as forming a continuous phase of plasticized film [48]. The homogenous structure of various films demonstrates the conspicuous compatibility of the two polymers and a tight structure deficient in phase separation. In the current work, it has been found that cross-linkers could further change the interactions of matrix molecules within the films. In addition to bacterial cellulose, the composite films present a clearer fiber structure. With the addition of borate and tripolyphosphate, the films were characterized by a compact, uniform, and homogeneous structure, indicating a closer interaction of matrix molecule induced by cross-linkers. A similar phenomenon has also been observed in previous studies [23].

### 3.3. Thermal Studies

**Figure 3:**
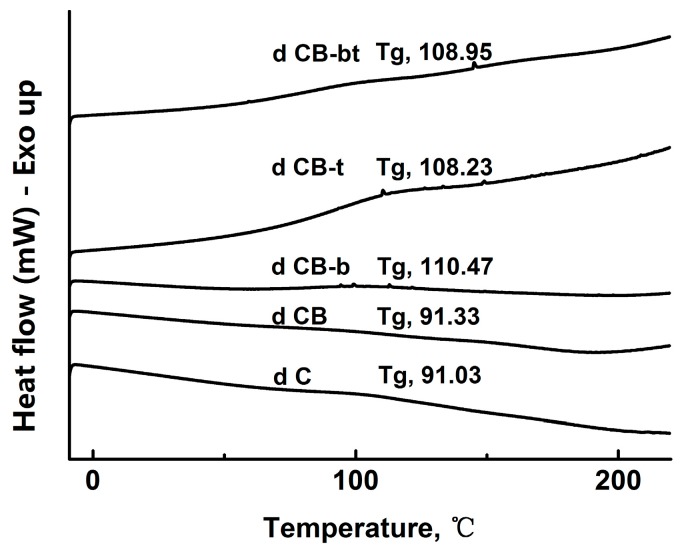
DSC thermograms for cast films C, CB, CB-b, CB-t, and CB-bt.

The thermal stability of biomaterials was determined using DSC. The thermograms for bacterial cellulose/chitosan 1/32-based films are shown in Figure 3. Glass transitions were observed at 91.03, 91.33, 110.47, 108.23, and 108.95 for C, CB, CB-b, CB-t, and CB-bt, respectively. Kadam et al. (2018) have reported that the mixing of polyphenolic cross-linkers could effectively increase the glass transitional temperature of chitosan films [49], which was in agreement with the results in the current work. We inferred that cross-linking could enhance the molecular interaction among the matrix polymers, and thus improve the thermal stability of bacterial cellulose/chitosan films. The hypothesis is to be further studied in the following section using FTIR. Only one *T*_g_ characteristic curve is detected for all samples, implying the entire compatibility of chitosan and bacterial cellulose. Inconsistent with the results of other studies [50], the broad endothermic peak could not be observed at approximately 110–120 °C in all samples, probably caused by the dissociation process of interchain hydrogen bonding of chitosan. Since a rapid drying method at a higher temperature was used to prepare the sample films, most of the polymers in the matrices were in an amorphous state [51], resulting in a different molecular aggregation state and configuration of chitosan in the films.

In addition, the sequence of *T*_g_ for all samples ranked as CB-b > CB-bt > CB-t > CB > C, indicating the degrees of difference in terms of the impact on the matrix molecular interaction by cross-linkers. *T*_g_ of the mixture is influenced by multiple factors, such as molecular weight [52], intermolecular interaction [53], compound compatibility [54], etc. Based on the complicated microcosmic matrix situation induced by different cross-linking methods, much more work is still needed to clarify such a thermo dynamic phenomenon. Nevertheless, it was interesting to find that the features of chitosan-based films, i.e., *T*_g_, hydrogen bond strength, surface hydrophobicity, and antimicrobial capacities, were clearly interrelated, as shown in the following section.

### 3.4. Infrared Spectroscopy

**Figure 4:**
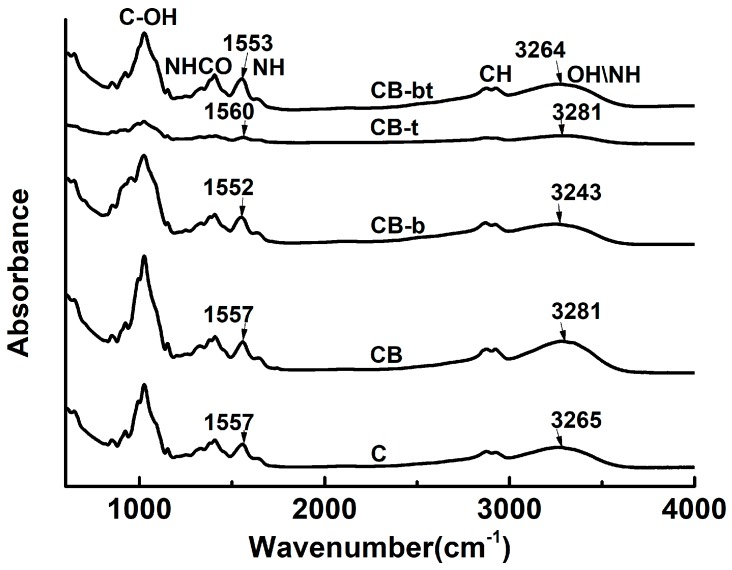
IR spectra of C, CB, CB-b, CB-t, and CB-bt.

FTIR spectra were used for a more profound exploration of the molecular interactions among the chitosan/bacterial cellulose matrix polymers. The IR absorbance of C, CB, CB-b, CB-t, and CB-bt is shown in Figure 4. The peak above 3000 cm^−1^ corresponds to the combined stretching of hydroxyl [55]. Hydroxyl groups in a carbohydrate matrix can exist in the form of hydrogen, bonded or free, without being implicated in any hydrogen bonds [56]. The vibration frequency of both can be attributed to the range 3000–3600 cm^−1^, while the characteristic IR band of free hydroxyls distributes in the higher frequency region [57]. Hence, as the hydrogen boned hydroxyls turn into free hydroxyls, the peak above 3000–3600 cm^−1^ shifts to a higher frequency range.

A strong switch of the characteristic absorption band of the hydrogen bond is measured as 3265, 3281, 3243, 3281, and 3264 cm^−1^ for C, CB, CB-b, CB-t, and CB-bt, respectively, indicating that the electrostatic interaction between hydroxyl and borate induces a stronger hydrogen bond network in chitosan/bacterial cellulose films. Furthermore, a band measured at 1557 cm^−1^ (CB) characteristic as amide (Ⅱ) [8] shifts to the lower wavenumber at 1552 cm^−1^ (CB-b), conforming to the intensified intermolecular interaction for the hydroxyl group induced by borate. Inconsistent with other results by Benucci et al. [58], a decrease of the amide (Ⅱ) signal was not detected, and it was speculated that the non-covalent cross-linking, caused by tripolyphosphate in the current work, did not lead to the reduction of hydrogen in the group. Meanwhile, it has been found that the rank of hydrogen bond strength followed the same order with that of *T*_g_, i.e., CB-b > CB-bt ≥ CB-t ≥ CB, indicating that the intermolecular force was one of the main forces that modulated the matrix thermodynamic properties.

### 3.5. Contact Angle

The contact angle with the film surface was measured to monitor the hydrophobic properties of produced matrices. Observed from the spatially varied curves of drips on the films, the contact angles were measured and the average was tabulated (Table 1). The contact value followed a rank of CB-b > CB-bt > CB-t > C> CB. Meanwhile, moisture absorption was found to decrease the surface contact angle for all tested films, according to the comparison of results of dry and wet films. The addition of cross-linkers, particularly borate, induced a higher contact angle for all tested samples, implying that the cross-linkers used in the current work might promote the hydrophobic properties of the film surface.

It is intriguing to note that the values of the film contact angle are in agreement with the reverse order sequence of the chitosan/bacterial cellulose-based matrix hydrogen bond network detected by IR spectroscopy, i.e., CB ≥ CB-t > CB-bt >CB- b. The strength of the matrix hydrogen bond network seemed to have a direct relationship with the surface hydrophobic properties. A reasonable explanation for this is that the cross-linkers led to a decrease of the free polar chemical group in the film surface [59]. Thus, the water/matrix polymer interactions were reduced while the surface hydrophobicity was heightened [60].

### 3.6. Water Vapor Absorption, Swelling Degree and Water Activity

In spite of possessing quite a few merits of chitosan films, reduced mechanical properties is a main shortcoming, especially after wet absorption [61]. Thus, hygroscopicity is considered as one of the essential qualities of a chitosan film or coating [62]. In this respect, water vapor absorption (WVA), swelling degree (SD), and water activity (WA) values of all chitosan/bacterial cellulose films were measured and tabulated in Table 2. On the basis of a statistical analysis, it was indicated that the mode of cross-linking significantly affected the SD of films, yet the changes for WVA and SD were minor. Interestingly, it has been found that it was CB-b, the film with the strongest hydrogen bond network according to the results of IR, that possessed the lowest swelling degree, implying that the resistance to water absorption was due to the reduced existence of polar chemical groups and the decreased free volumes of polymer molecule relaxation. The water absorption of polymeric films was closely related to the hydrophobic or hydrophilic properties, along with the existence of free molecule volumes in the matrices [63]. It is speculated that cross-linking might reduce the interaction of water with the matrix molecule, and further influence the retention of water inside the films. The result was in good accordance with the values achieved by Vartiainen et al., who investigated the water absorption of chitosan composite films reinforced with nanoclay [64].

### 3.7. Antimicrobial Testing

**Figure 5:**
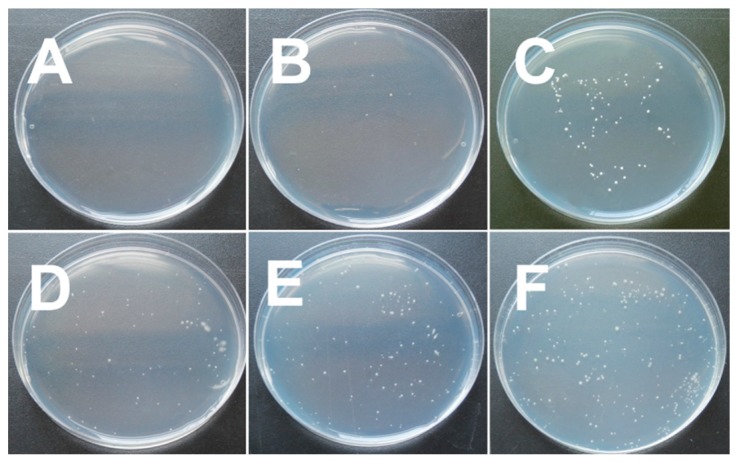
Images of antibacterial efficacy against *S. cerevisiae* for (**A**) C, (**B**) CB, (**C**) CB-b, (**D**) CB-t, (**E**) CB-bt, and (**F**) Blank (PE film).

In order to assess the antimicrobial properties of hybrid films, the *E. coli* (bacterial), *B. cinerea* (mold), and *S. cerevisiae* (yeast) were chosen as the representative microorganisms. Table 3 presents the antimicrobial results of C, CB, CB-b, CB-t, and CB-bt films. For the sake of reliability, the experiments were repeated three times. The pH of the bacterial culture medium was measured before and after the sample films were immersed, while no obvious change was found, probably due to the fact that most of the acetic acid volatilized in the sample preparation process. Apparently, the successive order of antimicrobial activity was shown as C ≥ CB > CB-b ≥ CB-t > CB-bt. As was mentioned in the literature, chitosan is antimicrobial against a wide variety of microbes, which originates from its polycationic character [65]. In this work, almost all *E. coli*, *B. cinerea*, and *S. cerevisiae* treated with C and CB were killed (Figure 5 shows the results of anti *S. cerevisiae* as representative). Unexpectedly, our results showed that the addition of cross-linkers remarkably weakened the antimicrobial ability of CB films.

The antimicrobial mechanism of chitosan is correlated with the cell death generated from the disruption of cell functioning or the destruction of the cell wall caused by the electrostatic interactions among cationic amino groups (NH_3_^+^) in chitosan molecules and anionic teichoic acids in the cell wall [66]. In our study, it was speculated that borate and tripolyphosphate could effectively disturb the interaction of chitosan with microbes by changing the ionic properties of amino groups, thus decreasing the efficacy of chitosan against tested *E. coli*, *B. cinerea*, and *S. cerevisiae*. Though cross-linkers have been widely used in enhancing the mechanical qualities of chitosan films [20], to the best of our knowledge, their impact on the antimicrobial effect has rarely been reported. In the current work, a critical issue has been put forward and investigated, i.e., certain cross-linkers actually significantly decrease the antimicrobial activity of chitosan.

## 4. Conclusions

This study focused on expounding the impact of cross-linking mode on the physical and antibacterial properties of chitosan/bacterial cellulose composite films. It has been found that the cross-linking mode microscopically affects the molecular interaction and microstructure, and macroscopically leads to changes in the films’ mechanical and thermodynamic properties, hydrophobicity, and water absorption. It is also worth noting that cross-linking can reduce the bacteriostatic function of chitosan/bacterial cellulose composite films. The results of this work prove that due to the varied intermolecular interactions between the cross-linkers and matrix polymers in the matrices, cross-linking mode has a significant impact on the practicability of the composite packaging film.

## Figures and Tables

**Table 1:** The impact of cross-linking treatment on the contact angle of dry and wet films from chitosan (C), 1/32 bacterial cellulose/chitosan (CB), and 1/32 bacterial cellulose/chitosan treated with borate (CB-b), tripolyphosphate (CB-t), or a mixture of borate and tripolyphosphate (CB-bt).

Sample	Contact Angle/°	
	DRY FILM	Wet Films
C	93.4 ± 0.8	88.8 ± 0.5
CB	91.2 ± 0.2	85.2 ± 1.1
CB-b	103.8 ± 1.3	101.3 ± 0.5
CB-t	94.7 ± 0.9	85.5 ± 0.4
CB-bt	99.4 ± 0.4	94.5 ± 0.1

**Table 2:** WVA, SD, and WA of C, CB, CB-b, CB-t, and CB-bt.

Samples	Water Absorption (×%)	Swelling Degree (×%)	Water Activity (Dry Films)
C	31.9 ± 0.5	71.7 ± 2.4	0.13 ± 0.01
CB	31.1 ± 0.3	42.0 ± 2.0	0.13 ± 0.01
CB-b	28.0 ± 1.7	20.7 ± 1.1	0.14 ± 0.01
CB-t	30.9 ± 1.5	29.5 ± 4.3	0.16 ± 0.01
CB-bt	29.7 ± 0.5	32.4 ± 1.5	0.14 ± 0.01

**Table 3:** A comparison of antimicrobial activity of C, CB, CB-b, CB-t, and CB-bt against *E. coli*, *B. cinerea*, and *S. cerevisiae*.

Samples	Bacteria Inhibitory Rate (×%)	Mold Inhibitory Rate (×%)	Yeast Inhibitory Rate (×%)
C	92.4 ± 2.9	72.8 ± 10.7	100.0 ± 0.0
CB	90.8 ± 6.4	77.8 ± 11.4	95.5 ± 6.4
CB-b	85.5 ± 4.7	51.0 ± 8.9	62.9 ± 2.3
CB-t	85.0 ± 3.7	51.5 ± 18.6	65.9 ± 0.3
CB-bt	73.1 ± 2.7	36.9 ± 14.7	52.8 ± 19.6
